# Effects of Karela (*Bitter Melon; Momordica charantia*) on genes of lipids and carbohydrates metabolism in experimental hypercholesterolemia: biochemical, molecular and histopathological study

**DOI:** 10.1186/s12906-017-1833-x

**Published:** 2017-06-17

**Authors:** Dalia Yossri Saad, Mohamed Mohamed Soliman, Ahmed A. Baiomy, Magdy Hassan Yassin, Hanan Basiouni El-Sawy

**Affiliations:** 1 Medical Laboratory Department, Faculty of Applied Medical Sciences, Taif University, Turabah, Saudi Arabia; 2 Biology Department, Faculty of Science, Cairo University, Cairo, Egypt; 3 Department of Biochemistry, College of Veterinary Medicine, Benha University, Moshtohor, P.O. 13736, Benha, Egypt; 4 Biology Department, Faculty of Science, Taif University, Turabah, Saudi Arabia; 5 Reproductive Diseases Department, Animal Reproduction Research Institute (ARRI), Al-Haram, Giza, Egypt; 6 Medical Microbiology Department, Faculty of Applied Medical Sciences, Taif University, Turabah, Saudi Arabia; 7 Department of Nutrition and Clinical Nutrition, Faculty of Veterinary Medicine, Kafrelsheikh University, Cairo, Egypt

**Keywords:** Carbohydrate, Gene expression, Hypercholesterolemia Karela, Lipids

## Abstract

**Background:**

Hypercholesterolemia is a serious diseases associated with type-2 diabetes, atherosclerosis, cardiovascular disorders and liver diseases. Humans seek for safe herbal medication such as karela (Momordica charantia/bitter melon) to treat such disorders to avoid side effect of pharmacotherapies widely used.

**Methods:**

Forty male Wistar rats were divided into four equal groups; control group with free access to food and water, cholesterol administered group (40 mg/kg BW orally); karela administered group (5 g /kg BW orally) and mixture of cholesterol and karela. The treatments continued for 10 weeks. Karela was given for hypercholesterolemic rats after 6 weeks of cholesterol administration. Serum, liver and epididymal adipose tissues were taken for biochemical, histopathological and genetic assessments.

**Results:**

Hypercholesterolemia induced a decrease in serum superoxide dismutase (SOD), catalase, reduced glutathione (GSH) and an increase in malondialdehyde (MDA) levels that were ameliorated by karela administration. Hypercholesterolemia up regulated antioxidants mRNA expression and altered the expression of carbohydrate metabolism genes. In parallel, hypercholesterolemic groups showed significant changes in the expression of PPAR-alpha and gamma, lipolysis, lipogenesis and cholesterol metabolism such as carnitine palmitoyltransferase-1 (CPT-1). Acyl CoA oxidase (ACO), fatty acids synthase (FAS), sterol responsible element binding protein-1c (SREBP1c), 3-hydroxy-3-methylglutaryl coenzyme A reductase (HMG-CoAR) and cholesterol 7α-hydroxylase (CYP7A1) at hepatic and adipose tissue levels. Interestingly, Karela ameliorated all altered genes confirming its hypocholesterolemic effect. Histopathological and immunohistochemical findings revealed that hypercholesterolemia induced hepatic tissue changes compared with control. These changes include cholesterol clefts, necrosis, karyolysis and sever congestion of portal blood vessel. Caspase-3 immunoreactivity showed positive expression in hepatic cells of hypercholesterolemic rats compared to control. All were counteracted and normalized after Karela administration to hypercholesterolemic group.

**Conclusion:**

Current findings confirmed that karela is a potential supplement useful in treatment of hypercholesterolemia and its associated disorders and is good for human health.

## Background

Metabolic syndrome has become the most prevalent worldwide epidemic diseases in recent decades. A recent national health survey conducted in mainland China revealed that 60 million people are obese and 200 million are overweight [1]. As known, liver is the functional tissue that controls the production of triglycerides (TGs) and glucose for use by other tissues, all are regulated by lipogenesis and gluconeogenesis [2]. Studies have demonstrated that excessive lipid accumulation in the liver is associated with oxidative stress and hepatic mitochondrial dysfunction [3, 4]. High-fat diet induced overproduction of reactive oxygen species in adipose tissue and liver [5].

Obesity, hypertriglyceridemia, and/or hypercholesterolemia are the common causes for many diseases such as cardiovascular [6] and liver diseases [7]. Rat fed with high cholesterol diet can be used as model of the human obesity syndrome [8]. The liver is the first organ to metabolize the ingested cholesterol and it is affected by oxidative stress that results from an imbalance between the production of free radicals and effectiveness of antioxidant systems [9]. Rats fed high cholesterol diet showed several abnormalities in liver sections such as cholesterol clefts, hepatotoxicity and fatty liver [10, 11].

Hypercholesterolemia, hypertension, disorders in glucose metabolism, smoking, aging, and social stress are the main risk factors for cardiovascular diseases [12]. Studies conducted showed that the incidence of cardiovascular events increased with increasing serum cholesterol levels [13]. Therefore, the normalization of serum cholesterol levels is important for preventing cardiovascular diseases and its associated disorders and alteration in lipids and carbohydrate metabolism. Lowering of serum lipid levels through dietary or drug therapy seems to be associated with a decrease in the risk of vascular disease and related complications [14, 15].

Karela, the fruit of which is known as *Momordica charantia*, bitter gourd or bitter melon, is a common edible vegetable in Asia. Approximately 93.2% of karela is water, and protein and lipids account for 18.02% and 0.76% of its dried weight, respectively [16]. Physiological benefits, including hypoglycemia, hypolipidemia, anti-virus and anti-carcinogenic effects have been reported [17, 18]. It has been shown that karela reduced high fat diet induced obesity, hyperlipidemia, hyperglycemia, insulin resistance, and fatty liver in mice [19]. Karela has been used for the treatment of diabetes throughout the world [20, 21].

Karela’s hypoglycemic effect has been demonstrated in type 1 and type 2 diabetic rodents [22, 23]. Also, it decreases the levels of total cholesterol (TC), triglycerides, and phospholipids in streptozotocin-induced diabetic rats [24]. Phytochemical studies revealed the presence of alkaloid, flavonoids, sterols, anthraquinones, and phenols, which represented the main active components in karela leaves [25]. It has been found that the ethyl acetate extract of karela activates both PPARα and PPAR γ [26] which are ligand-activated transcription factors belonging to the nuclear receptor superfamily. They play a key role in the control of lipid and glucose homeostasis as transcriptional factors regulating genes encoding enzymes involved in these processes [27].

This study aimed to examine the effect of karela on experimental hypercholesterolemia at the biochemical, molecular and cellular levels using semi-quantitative PCR analysis and immunohistochemistry.

## Methods

### Materials and kits

Ethidium bromide and agarose were purchased from Sigma-Aldrich, St. Louis, MO, USA). The Wistar albino rats were purchased from King Fahd center for Scientific Research, King Abdel-Aziz University, Jeddah, Saudi Arabia. Serologic kits for HDLC, Cholesterol and triglycerides (TG) HUMAN Gesellschaft für Biochemica und Diagnostica mbH (Wiesbaden, Germany). The deoxyribonucleic acid (DNA) ladder was purchased from MBI, Fermentas, Thermo Fisher Scientific, USA. Qiazol for RNA extraction and oligo dT primer were purchased from QIAGEN (Valencia, CA, USA). Kits for antioxidants such as superoxide dismutase (SOD), catalase, reduced glutathione (GSH) and malondialdehyde (MDA) were bought from Bio-diagnostic Co., Dokki, Giza, Egypt.

### Animals and experimental design

Ethical Committee Office of the scientific Deans of Taif University, Saudi Arabia approved all procedures of this study for the project #4860–437-1. Forty male Wistar rats, 2 months old (200–222 g) were used for this study. Animals were kept under observation for 2 weeks to ensure complete acclimatization before the onset of the experiment. The animals were kept at equal light–dark cycle (12/12 h) with free access to food and water. Four groups each containing 10 healthy Wistar rats were used for the study as follows: Group 1, served as negative control with free access to food and water. Group 2 served as positive hypercholesterolemic group and was given orally cholesterol in a dose of 40 mg/kg body weight daily for 6 weeks. Group 3 was administered orally Karela in a dose of 5 g/kg body weight daily based on a previous study [28]. Group 4 administered orally cholesterol in a dose of 40 mg/kg body weight daily for 10 weeks plus karela in a dose of 5 g/ kg bw daily at week 6 and continued for 4 weeks later. Dose of cholesterol was used based on the finding of Co and To [29]. After 10 weeks, rats were inhaled dimethyl ether and decapitated after overnight fasting. Liver and adipose tissue were preserved in Bouin’s solution for histopathological examination and in Qiazol reagent for RNA extraction for gene expression.

### Karela preparation and administration

Fresh karela fruits (*Bitter melon*) was purchased from commercial local markets in Taif governate (Panda, Taif), Saudi Arabia. The plant fruits was identified by botanist in College of Science, Taif University, Saudi Arabia. Karela was washed thoroughly with water, and dried after cutting into small pieces, dried and powdered using a blender. The dose used was 5 g /kg BW by intragastric tube based on previous reports [28].

### Assay of biochemical parameters

Glucose was measured colormetrically using commercial available kits. Antioxidants such as superoxide dismutase, SOD, GSH, MDA and catalase were measured spectrophotometrically using commercial ELISA kits based on manufacturer’s instruction manual. Serum triacylglycerol, total cholesterol and high density lipoproteins-cholesterol (HDLC) were measured spectrophotometrically according to the manufacturer’s protocol.

### Histopathological and Immunohistochemical examination

The collected specimens of liver were fixed in 10% buffered neutral formalin solution and then routinely processed. Paraffin sections of 5 μm thickness were prepared, stained with Hematoxylin and eosin stain (H&E) as described before [30]. By using avidin-biotin-peroxidase method, the liver samples were embedded in paraffin and cut into 3 μm sections. Samples were mounted on positively charged slides for caspase 3 immunohistochemical examination. Sections were dewaxed, rehydrated and autoclaved at 95 °C for 20 min in antigen retrieval buffer (10 mM citrate buffer, pH 6). After washing with phosphate buffered saline (PBS), endogenous peroxidase was blocked using 3% H_2_O_2_ in methanol for 15 min. A primary rat specific antibody for caspase 3 (cat.no. RB 1197 B0, B1; Thermo Fisher Scientific Inc) was diluted in PBS (1:100), and incubated for 30 min. The slides were then rinsed three times with PBS. Horseradish peroxidase conjugated goat anti mouse IgG secondary antibody (Cat # 32230; Thermo Fisher Scientific Inc.) was incubated for 30 min with tissue sections. Extra rinsing for 3 times with PBS was done. Samples were visualized after 10 min from adding metal enhanced diaminobenzidine (DAB) substrate (Sigma-Aldrich, St. Louis, MO, USA) as a working solution (Thermo Fisher Scientific Inc.) as stated before [31]. The immune reactivity score was used to evaluate the intensity of immunohistochemical staining and the proportion of the stained cells [31].

### RNA extraction, cDNA synthesis and RT-PCR analysis

Total RNA was extracted from liver and epididymal adipose tissue samples (50 mg per sample) as stated before [32]. In short, samples were flash frozen in liquid nitrogen and subsequently stored at −70 °C in 1 ml Qiazol (QIAGEN, Valencia, CA, USA). Frozen samples were homogenized using a Polytron 300 D homogenizer (Brinkman Instruments, Westbury, NY, USA). Then, 0.3 ml chloroform was added to the homogenate. The mixtures after shaking for 30 s, centrifuged at 4 °C and 16,400 x *g* for 15 min. The supernatant was transferred to new tubes. Equal volume of isopropanol was added to the samples and centrifuged at 4 °C and 16,400 x *g* for 15 min. The RNA pellets were washed with 70% ethanol, briefly dries up, and then dissolved in diethylpyrocarbonate (DEPC) water. RNA concentration and purity were determined spectrophotometrically at 260 nm. The RNA integrity was confirmed in 1.5% denaturated agarose gel stained with ethidium bromide. The ratio of the 260/280 optical density of all RNA samples was 1.7–1.9. For cDNA synthesis, a mixture of 3 μg total RNA and 0.5 ng oligo dT primer (Qiagen Valencia, CA, USA) in a total volume of 11 μl sterilized DEPC water was incubated in the Bio-Rad T100™ Thermal cycle at 65 °C for 10 min for denaturation. Then, 2 μl of 10X RT-buffer, 2 μl of 10 mM dNTPs and 100 U Moloney Murine Leukemia Virus (M-MuLV) Reverse Transcriptase (SibEnzyme. Ak, Novosibirsk, Russia) were added and the total volume was completed up to 20 μl by DEPC water. The mixture was then re-incubated in BIO-RAD thermal cycler at 37 °C for one hour, then at 90 °C for 10 min to inactivate the enzyme. For semi-quantitative RT-PCR analysis, specific primers for examined genes (Table 1) were designed using Oligo-4 computer program and synthesized by Macrogen (Macrogen Company, GAsa-dong, Geumcheon-gu. Korea). PCR was conducted in a final volume of 25 μl consisting of 1 μl cDNA, 1 μl of 10 pM of each primer (forward and reverse), and 12.5 μl PCR master mix (Promega Corporation, Madison, WI, USA), the volume was brought up to 25 μl using sterilized, deionized water. PCR was carried out using Bio-Rad T100™ Thermal Cycle machine with the cycle sequence at 94 °C for 5 min one cycle, followed by variable cycles (Table 1) each of which consists of denaturation at 94 °C for one minute, annealing at the specific temperature corresponding to each primer (Table 1) and extension at 72 °C for one minute with an additional final extension at 72 °C for 7 min. As a reference, expression of glyceraldehyde-3-phosphate dehydrogenase (G3PDH) mRNA was examined (Table 1). PCR products underwent electrophoresis on 1.5% agarose (Bio Basic, Markham, ON, Canada) gel stained with ethidium bromide in TBE (Tris-Borate-EDTA) buffer. PCR products were visualized under UV light and photographed using gel documentation system. The intensities of the bands from four different rats per group and three independent experiments were quantified densitometrically using Image J software version 1.47 (https://imagej.en.softonic.com/).

**Table 1:** PCR conditions and primer sequence for examined genes

mRNA expression	Forward primer (5′-3′)	Reverse primer (5′-3′)	PCR cycles and Annealing
PEPCK (236 bp)	TTTACTGGGAAGGCATCGAT	TCGTAGACAAGGGGGCAC	30 cycles, 52 °C 1 min
PK (229 bp)	ATTGCTGTGACTGGATCTGC	CCCGCATGATGTTGGTATAG	30 cycles, 52 °C 1 min
ACO (633 bp)	GCCCTCAGCTATGGTATTAC	AGGAACTGCTCTCACAATGC	35 cycles, 52 °C 1 min
CPT-1 (628 bp)	TATGTGAGGATGCTGCTTCC	CTCGGAGAGCTAAGCTTGTC	35 cycles, 52 °C 1 min
PPAR γ (550 bp)	CATTTCTGCTCCACACTATGAA	CGGGAAGGACTTTATGTATGAG	33 cycles 52 °C 1 min
PPAR-α (680 bp)	GAGGTCCGATTCTTCCACTG	ATCCCTGCTCTCCTGTATGG	35 cycles, 58 °C 1 min
FAS (345 bp)	CCAGAGCCCAGACAGAGAAG	GACGCCAGTGTTCGTTCC	37 cycles, 61 °C 45 s
SREBP-1c (191 bp)	GGAGCCATGGATTGCACATT	AGGAAGGCTTCCAGAGAGGA	35 cycles, 58 °C 50 s
HMG-CoAR (467 bp)	CCTGCTGCCATAAACTGGAT	GCCATTACAGTGCCACACAC	31 cyc les58°C 1 min
GST (575 bp)	GCTGGAGTGGAGTTTGAAGAA	GTCCTGACCACGTCAACATAG	35 cycles, 55 °C 1 min
CYP7A1 (574 bp)	CCTCCTGGCCTTCCTAAATC	GTACCGGCAGGTCATTCAGT	30 cycles 58 °C 1 min
SOD (410 bp)	AGGATTAACTGAAGGCGAGCAT	TCTACAGTTAGCAGGCCAGCAG	33 cycles, 55 °C 1 min
GAPDH (309 bp)	AGATCCACAACGGATACATT	TCCCTCAAGATTGTCAGCAA	25 cycles, 52 °C 1 min

#### Statistical analysis

Data are expressed as Means ± standard error (SE). Data were analyzed using analysis of variance (ANOVA) and post hoc descriptive tests by SPSS software version 11.5 for Windows (SPSS, IBM, Chicago, IL, USA).with *P* < 0.05 regarded as statistically significant. Regression analysis was performed using the same software.

## Results

### Effects of karela on changes in antioxidants induced by hypercholesterolemia

Table 2 shows that experimental hypercholesterolemia was associated with an increase in serum total cholesterol levels, triglycerides and glucose. In parallel there was a decrease in high density lipoproteins (HDL). Administration of Karela normalized and ameliorated this altered parameters and increased HDL levels (Table 2). Hypercholesterolemia as seen in Table 3, significantly increased the serum levels of malondialdehyde (MDA) and decreased serum levels of super oxide dismutase (SOD), reduced glutathione (GSH) and catalase. Administration of karela significantly improved the decrease in antioxidant activity and normalized the increase in MDA (Table 3). Of note, administration of karela to control rats induced high antioxidants potency.

**Table 2:** Protective effects of Karela on hypercholesterolemia induced changes in serum lipid levels in Wistar rats

	HDLC (mg/dL)	Cholesterol (mg/dL)	TG (mg/dL)	Glucose (mg/dL)
Control	30.8 ± 0.6	63.3 ± 4.1	30.5 ± 4.2	84.5 ± 2.3
Cholesterol	19.4 ± 0.7*	180.5 ± 6.6*	99.8 ± 7.4*	89 ± 10.2*
Karela	34.5 ± 0.9	54.5 ± 2.25	30.6 ± 1.78	76 ± 8.7
Karela + Cholesterol	38.32 ± 2.4#	81.2 ± 7.5#	55.3 ± 5.5#	69 ± 13.1#

Values are means ± standard error (SEM) for 10 different rats per each treatment. Values are statistically significant at *p < 0.05 Vs. control and #*p* < 0.05 Vs. cholesterol administered rats

**Table 3:** Protective effects of Karela extract on hypercholesterolemia induced changes in antioxidants levels in Wistar rats

	SOD (U/ml)	Catalase (U/ml)	GSH (mg/dl)	MDA (nmol/ml)
Control	347.9 ± 37.5	32.5 ± 4.4	0.4 ± 0.01	5.17 ± 0.3
Cholesterol	202.7 ± 25.4^*^	20.23 ± 2.8^*^	0.2 ± 0.02^*^	37 ± 4. 2^*^
Karela	566.3 ± 28.1^$^	31.80 ± 8.3	0.8 ± 0.02^$^	4. 6 ± 0.01
Karela + Cholesterol	286.7 ± 44.2^#^	66.5 ± 7.2^#^	h1.7 ± 0.07^#^	16.7 ± 1.02^#^

Values are means ± standard error (SEM) for 10 different rats per each treatment. Values are statistically significant at *p < 0.05 Vs. control, #*p* < 0.05 Vs. cholesterol administered rats and $*p* < 0.05 Vs. control

### Pathological and immunohistochemical changes in the liver after karela administration to hypercholesterolemic rats

**Fig. 1:**
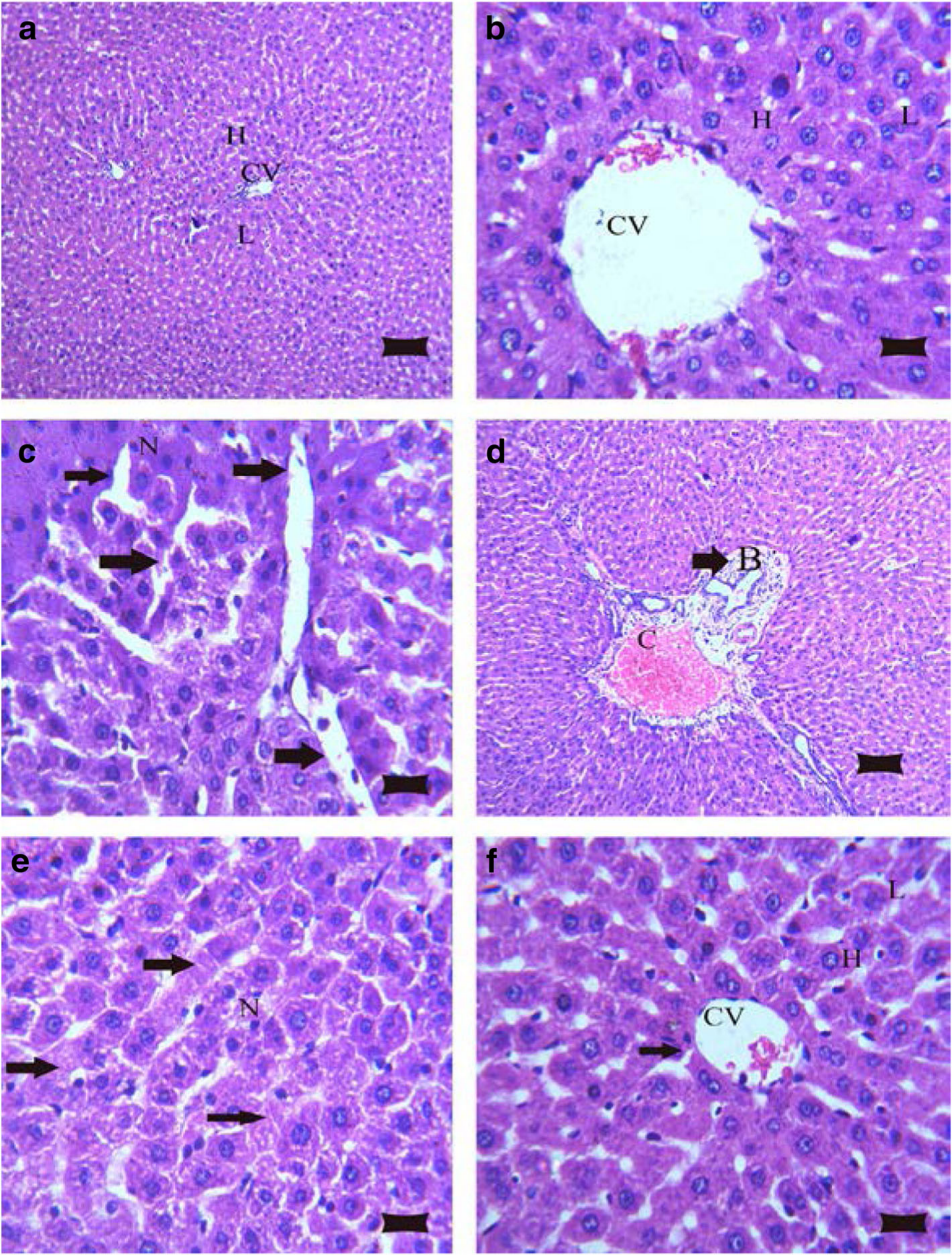
Photomicrograph of rat livers in the various groups: **a** Control group, showing the normal histological structure of hepatic lobule (*L*) with centrally located euchromatic nucleus of hepatocyte (*H*) surround central vein (*CV*) (H&E; bar, 89 μm); **b** Karela administered group, shows normal histological structure of hepatic lobule (*L*), hepatocyte (*H*) and central vein (*CV*) (H&E; bar, 14 μm); **c** Cholesterol group, showing deposition of cholesterol crystals in many radicular cysts forming cholesterol clefts (*arrows*) and necrosis (*N*) of hepatocytes (H&E; bar, 14 μm); **d** Cholesterol administered group, showing sever congestion of portal blood vessel (*C*) with extensive portal fibrosis (*arrow*) and hyperplasia of epithelial lining of the bile duct (*B*) (H&E; bar, 89 μm); **e** Cholesterol group, showing necrosis in hepatocytes (*N*) with karyolysis of hepatocytes nuclei (*arrows*) (H&E; bar, 14 μm); and **f** Cholesterol and Karela group, show apparent normal hepatic parenchyma with few cholesterol clefts (*arrow*), hepatic lobule (*L*), hepatocyte (*H*), central vein (*CV*) (H&E; bar, 14 μm)

**Fig. 2:**
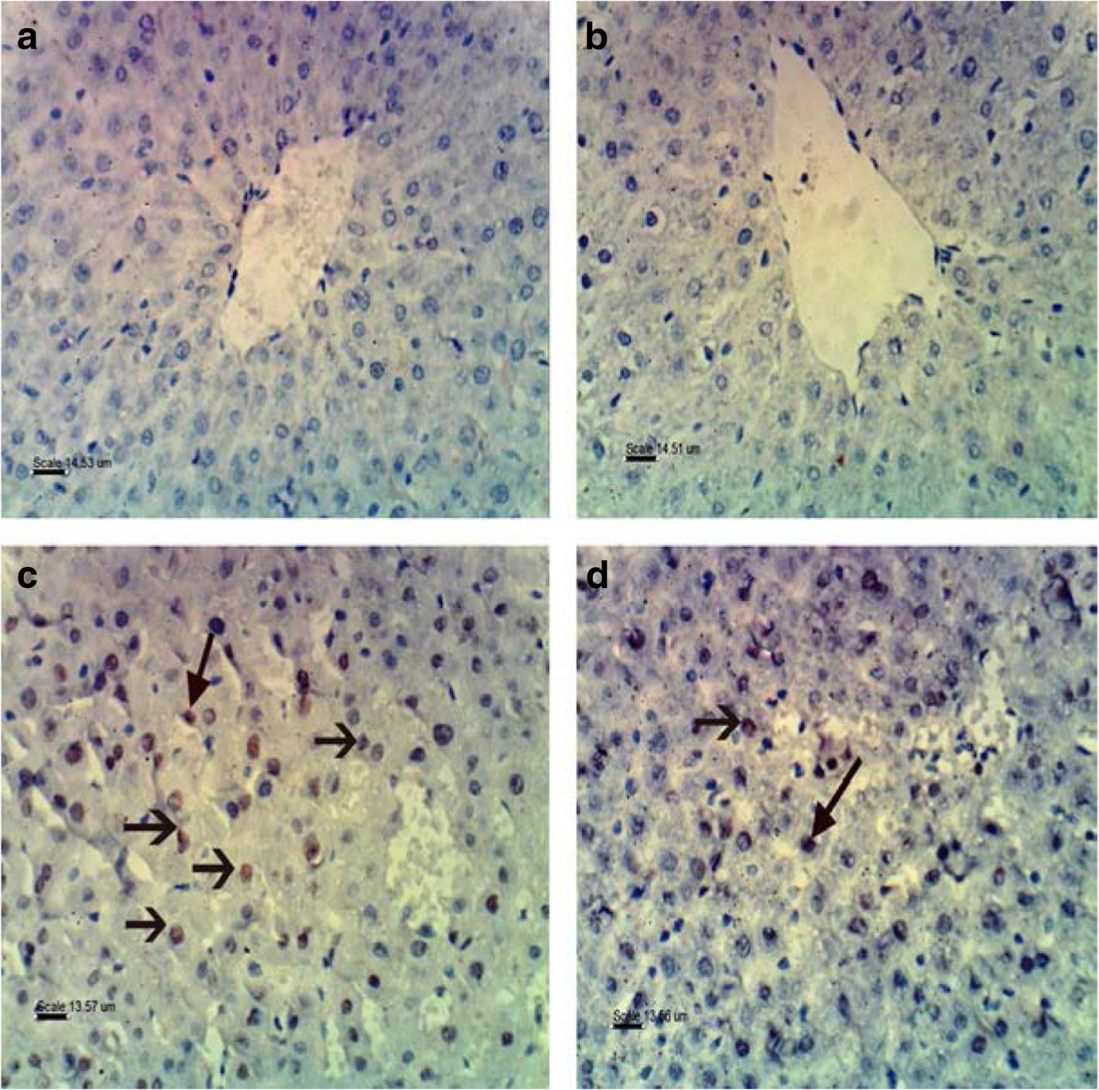
Photomicrograph of immunohistochemical staining of Caspase 3 in liver of rat in examined groups: **a** Control group, showing negative immunohistochemical staining of caspase 3 immunoreactivity (bar, 14.53 μm); **b** Karela administered rats, showing negative immunohistochemical staining of Caspase 3 (bar, 14.51 μm); **c** Cholesterol administered group, showing strong immunohistochemical staining and immune reactivity of Caspase 3 (bar, 13.57 μm); **d** Cholesterol and Karella treatment group, showing mild immunohistochemical staining of Caspase 3 (bar, 13.56 μm)

The histological examination of liver in control and karela administered rats had a normal architecture of hepatic lobules with plates of polygonal hepatocytes radially arranged around the central vein and separated by blood sinusoids, the hepatocytes revealed an acidophilic cytoplasm and rounded nuclei (Fig. 1a, b). In hypercholesterolemic rats revealed loss of normal hepatic architecture with deposition of cholesterol crystals are found in many radicular cysts forming cholesterol clefts and necrosis of hepatocytes (Fig. 1c). Also, sever congestion of portal blood vessel with extensive portal fibrosis, hyperplasia of epithelial lining of the bile duct and karyolysis of hepatocytes nuclei (Fig. 1d, e). In liver of hypercholesterolemic rats administered karela, liver showed apparent normal hepatic parenchyma with few cholesterol clefts (Fig. 1f). In Immunohistochemical examination for caspase-3 expression in liver. The control and karela administered rats showed negative immunohistochemical staining for caspase-3 immunoreactivity (Fig. 2a, b). Liver of hypercholesterolemic rats showed strong immunohistochemical staining of caspase 3 (Fig. 2c). Liver of hypercholesterolemic rats administered karela showed mild immunohistochemical staining of Caspase-3 (Fig. 2d).

### Effects of karela on gene expression of antioxidants and carbohydrate associated genes altered by hypercholesterolemia

**Fig. 3:**
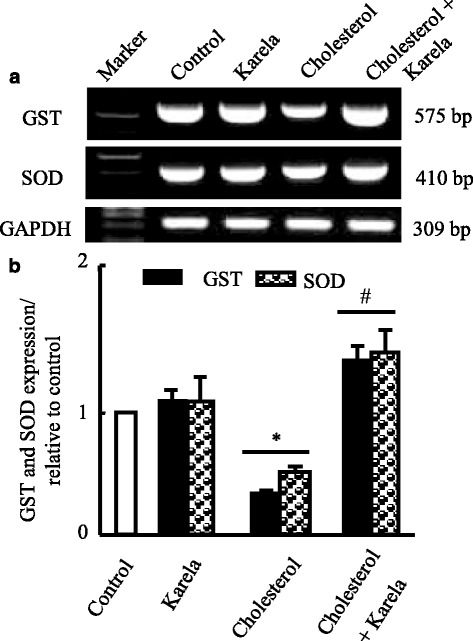
Protective effect of karela on changes in antioxidants expression induced by hypercholesterolemia in liver. Hypercholesterolemic rats were administered karela for 4 consecutive weeks. Total RNA was extracted from liver tissues and the expressions of GST and SOD were analyzed by semi-quantitative RT-PCR analysis. Values are means ± SE of 10 rats. ^*^ *P* < 0.05 Vs control group; ^#^ *P* < 0.05 VS hypercholesterolemic group. *Upper panels* (**a**) are mRNA expression of examined genes. *Lower columns* (**b**) are densitometric analysis of gene expression​ for *upper panels*

**Fig. 4:**
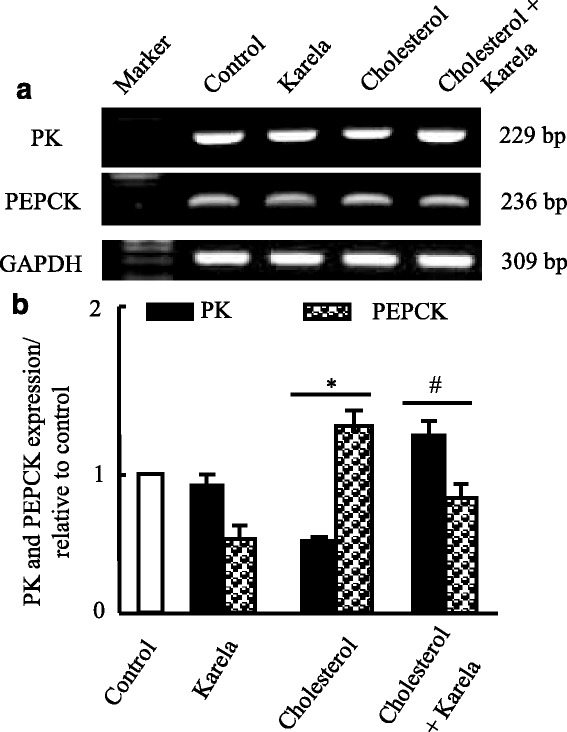
Protective effect of karela on changes in PK and PEPCK expression induced by hypercholesterolemia in liver. Hypercholesterolemic rats were administered karela for 4 consecutive weeks. Total RNA was extracted from liver tissues and the expressions of PK and PEPCK were analyzed by semi-quantitative RT-PCR analysis. Values are means ± SE of 10 rats. ^*^ *P* < 0.05 Vs control group; ^#^ *P* < 0.05 VS hypercholesterolemic group. *Upper panels* (**a**) are mRNA expression of examined genes. *Lower columns* (**b**) are densitometric analysis of gene expression​ for *upper panels*

Figure 3, shows that hypercholesterolemia decreased significantly glutathione-S-transferase (GST) and superoxide dismutase mRNA expression in liver. Administration of karela for cholesterol administered rats normalized mRNA expression pattern compared to control rats. On the other hand, hypercholesterolemia decreased pyruvate kinase (PK) mRNA expression that were ameliorated when karela co-administered for hypercholesterolemic rats (Fig. 4). In contrast, karela down regulated phosphoenolpyruvate carboxykinase (PEPCK) that was upregulated in hypercholesterolemic rats (Fig. 4). Co-administration of karela with cholesterol ameliorated this increase in PEPCK expression in hypercholesterolemic rats.

### Effects of karela on lipolysis and lipogenesis gene expression altered by hypercholesterolemia

**Fig. 5:**
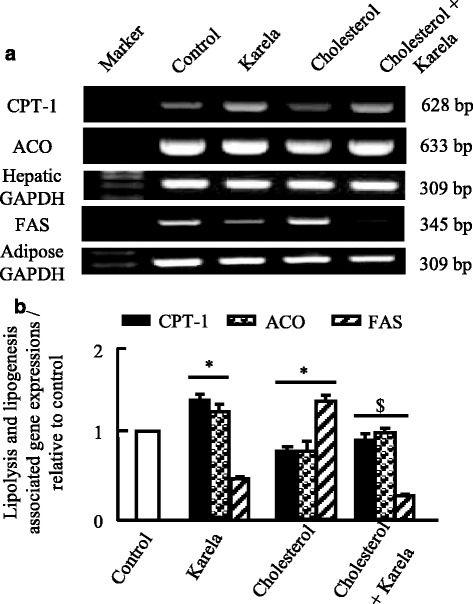
Protective effect of karela on changes in CPT-1, ACO and FAS expression induced by hypercholesterolemia in liver and adipose tissue. Hypercholesterolemic rats were administered karela for 4 consecutive weeks. Total RNA was extracted from liver tissues and the expressions of CPT-1, ACO and FAS were analyzed by semi-quantitative RT-PCR analysis. Values are means ± SE of 10 rats. ^*^ *P* < 0.05 Vs control group; ^#^ *P* < 0.05 VS hypercholesterolemic group. *Upper panels* (**a**) are mRNA expression of examined genes. *Lower columns* (**b**) are densitometric analysis of gene expressions​ for *upper panels*

**Fig. 6:**
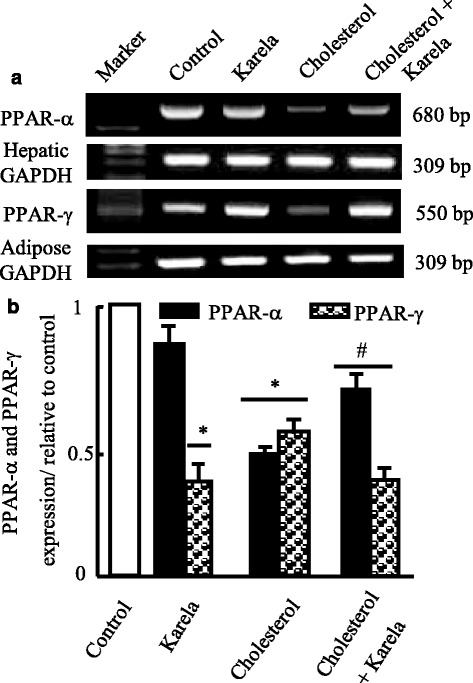
Protective effect of karela on changes in PPAR-α and PPAR-γ expression induced by hypercholesterolemia in liver and adipose tissue. Hypercholesterolemic rats were administered karela for 4 consecutive weeks. Total RNA was extracted from liver tissues and the expressions PPAR-α and PPAR-γ were analyzed by semi-quantitative RT-PCR analysis. Values are means ± SE of 10 rats. ^*^ *P* < 0.05 Vs control group; ^#^ *P* < 0.05 VS hypercholesterolemic group. *Upper panels* (**a**) are mRNA expression of examined genes. *Lower columns* (**b**) are densitometric analysis of gene expression for *upper panels*

To examine the expression of genes for fatty acids oxidation such as acyl CoA oxidase (ACO) and carnitine palmitoyltransferase-1 (CPT-1), RT-PCR was carried done on liver tissues. Figure 5, shows that karela alone partially increased mRNA expression of ACO and CPT-1 while cholesterol administered rats showed downregulation in their mRNA expression. When karela co-administered with cholesterol it ameliorated this downregulation. In parallel, the enzyme essential for fatty acids synthesis (fatty acids synthase; FAS) showed a decrease in karela administered rats and upregulated in hypercholesterolemic rats. It was downregulated when karela co-administered with cholesterol (Fig. 5). Next, we examined the expression of PPAR-α and PPAR-γ in the liver and adipose tissue respectively as a transcriptional regulator of lipid metabolism and glucose homeostasis. As seen in Fig. 6, Karela activated PPAR-α and PPAR-γ expression in liver and adipose tissue respectively that were downregulated significantly in hypercholesterolemic groups.

### Effects of karela on genes regulate cholesterol metabolism expression

**Fig. 7:**
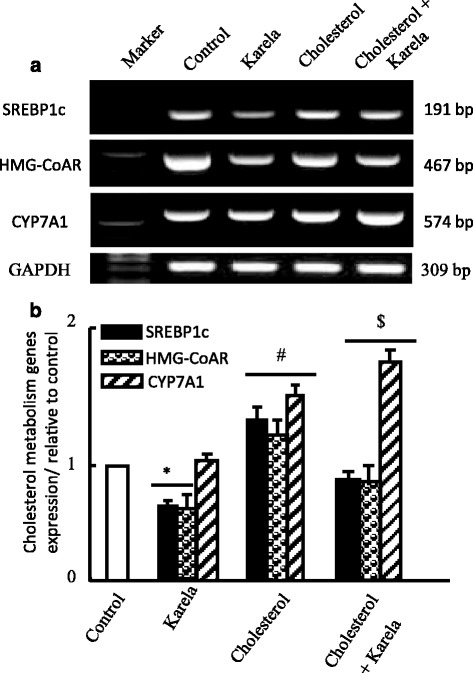
Protective effect of karela on changes in SREBP1c, HMG-CoAR and CYP7A1 expression induced by hypercholesterolemia in liver. Hypercholesterolemic rats were administered karela for 4 consecutive weeks. Total RNA was extracted from liver tissues and the expressions of SREBP1c, HMG-CoAR and CYP7A1 were analyzed by semi-quantitative RT-PCR analysis. Values are means ± SE of 10 rats. ^*^ *P* < 0.05 Vs control group; ^#^ *P* < 0.05 VS karela group and ^$^ *P* < 0.05 Vs hypercholesterolemic group. *Upper panels* (**a**) are mRNA expression of examined genes. *Lower columns* (**b**) are densitometric analysis of gene expression for *upper panels*

Finally, we examined the effect of karela on genes associated with cholesterol metabolism such as sterol responsible element binding protein-1c (SREBP-1c), 3-hydroxy-3-methylglutaryl coenzyme A reductase (HMG-CoAR) and cholesterol 7α-hydroxylase (CYP7A1). Karela alone showed downregulation in SREBP-1c and HMG-CoAR expression that were upregulated in cholesterol administered rats (Fig. 7). Administration of karela to hypercholesterolemic rats normalized significantly this increase in mRNA expression of SREBP-1c and HMG-CoAR. Regarding the expression of CYP7A1, Karela showed partial increase in CYP7A1 and feeding cholesterol alone induced more and clear stimulatory effect in CYP7A1 mRNA compared to karela. When karela administered to hypercholesterolemic rats an additive upregulation effect was reported (Fig. 7).

## Discussion

The present study interpret that karela has hypocholesterolemic effect through the reduction of hepatic oxidative stress and apoptosis, regulation of genes associated with glucose, lipid and cholesterol metabolism at biochemical, cellular and molecular levels. Hypercholesterolemia showed liver affection that presented by apoptosis. In liver diseases, cell repair, inflammation, regeneration, and fibrosis may all be triggered by apoptosis [33]. The liver is the first organ to metabolize the ingested cholesterol and it is affected by oxidative stress that results from an imbalance between the production of free radicals and effectiveness of antioxidant defense systems [9]. The present data revealed that rats fed high cholesterol diet had abnormalities in liver sections such as cholesterol clefts, necrosis of hepatocytes and congestion of portal blood vessel. Previous reports showed that high cholesterol diet causes hepatotoxicity and fatty liver [10, 11] and increased apoptotic hepatocytes number [34]. The potential mechanisms for the beneficial effects of karela on fatty liver involve reducing inflammation, eliminating oxidative stress, and suppressing apoptosis as confirmed here and in another study [19].

As known Chen and his team [35], are the first that confirmed the anti-adiposity effect of karela, who subsequently showed a decrease triglycerides contents of liver cells and muscle in rats fed high fat diet containing freeze-dried bitter melon juice [36]. Recently, adipose tissue has been recognized to serve as an energy storage, and an endocrine organ by releasing adipokines into the circulation to regulate both adipose tissue mass and the functions of other tissues by affecting systemic lipid and glucose metabolism [37]. The antiobesity effect of bitter melon was confirmed [38]. Here, hypercholesterolemia caused oxidative stress and decreased antioxidants levels and expression in liver, and karela ameliorated this alterations. In parallel, Wu and Ng [39] found that extracts of bitter gourd grown in Taiwan, possessed higher antioxidant and free radical-scavenging activities.

As known liver gluconeogenesis constitutes about 60–97% of the hepatic glucose production. PEPCK is a key rate-limiting enzyme of gluconeogenesis. High fat diet consumption can upregulate PEPCK expression in mice [40]. In our present study, the PEPCK expression increased in hypercholesterolemic rats. Administration of karela restored PEPCK expression to a level similar to control group. Therefore, karela induced in hypercholesterolemic rats hypoglycemic effect by inhibiting hepatic glucose production via a decrease in PEPCK expression and without effect on PK mRNA (Fig. 4). Possibly, the hypoglycemic effect of karela is due to inhibition of glucose-6-phosphatase activity [41].

Nerurkar and his colleagues [42] reported in vitro that karela inhibited human preadipocytes differentiation through down regulation in PPAR-γ, SREBP-1c, resistin and upregulation in lipolysis. Among the factors that affect lipogenesis are peroxisome proliferator activator receptor-γ (PPAR-γ) and SREBP-1c. PPAR-γ is the master regulator of adipogenesis [43], while SREBP-1c is an adipogenic transcription factors [44]. The balance between adipogenesis and lipolysis is critical for the proper function of adipose tissue, which consecutively affects the pathogenesis of obesity and its associated metabolic functions (42). To treat obesity, you need multiple interventions such as exercise programs, diet, behavioral modification and pharmacotherapy. Karela showed clear results on lipid metabolism through regulation of the key enzymes essential for lipogenesis and lipolysis. It downregulated the expression of FAS and increased the expression of ACO and CPT-1. Our findings that karela suppressed FAS and SREBP1c gene expression postulate that karela might antagonize the transcriptional activity of lipogenic factors such as ADD1/SREBP-1c [44]. SREBP1c is a regulator of lipid homeostasis, lipogenesis and sterol biosynthesis. In our results SREBP1c was decreased by karela and increased in hypercholesterolemic rats and normalized when co-administered together. However, Huang et al. [45], did not observe any effects on SREBP-1c mRNA expression in the adipose tissue of rats fed high fat diet and karela. They suggested that karela possibly act at the protein levels or post-transcriptionally to affect these genes [45].

Cholesterol homeostasis is achieved through the regulation of cholesterol biosynthesis, the conversion of cholesterol to bile acids, and their excretion. Cholesterol homeostasis are regulated by HMG-CoAR and CYP7A1 [46]. HMG-CoAR is the rate-limiting enzyme in the synthesis of cholesterol, whereas CYP7A1 is the rate-limiting enzyme in the synthesis of bile acids from cholesterol via the classical pathway [47] Furthermore, CYP7A1 is partially regulated at the transcriptional level by the hepatic liver X receptor-α (LXRα) and farnesoid X receptor [48, 49]. LXRα is a transcription factor activated by the oxidized forms of cholesterol, serving as sensor of excessive intracellular cholesterol accumulation [48]. Farnesoid X receptor is a bile acid receptor and acts as the major hepatic bile acid sensor that regulate bile acid synthesis and transport. Moreover, more than 95% of the bile acids is reabsorbed in the distal ileum and carried back to the liver the body. Thus, hepatic LXRα and FXR play an important role in regulating cholesterol homeostasis through modulation of the biosynthesis of bile acids. Compared to hypercholesterolemic rats, we reported that Karela group increased CYP7A1 gene expression (Fig. 7) and decreased the serum TC level (Table 2). These results suggest that karela exerts its hypocholesterolemic activity by decreasing the reabsorption of bile acids in the intestine and facilitating the conversion of cholesterol to bile acids via up-regulation of CYP7A1 [28]. Moreover, Matsui et al., [28] concluded that the upregulation in CYP7A1 in karela administered groups is independent on LXR-α expression in examined hepatic tissues. Karela altered the HMGR mRNA level (Fig. 7), suggesting that the decreased serum cholesterol level in the karela group is dependent on regulation of hepatic cholesterol synthesis.

Finally, it can be concluded from previous reports that karela suppressed the effect of small heterodimer partner (SHP) that is implicated in bile acids biosynthesis, and this suppression enhanced the action of CYP7A1 through liver receptor homologue-1 (LRH-1). Therefore, this increase in CYP7A1 is the cause for the decrease in serum cholesterol levels (increase catabolism of blood cholesterol to bile acids in the liver) as reported in this study.

## Conclusion

This study demonstrated that karela altered hepatic and adipose tissue expression of genes associated with lipids and carbohydrate metabolism to improve blood cholesterol levels and hypercholesterolemia. It ameliorated the alteration induced by cholesterol on hepatic cell architecture, apoptosis and oxidative stress. Furthermore, our study showed that the karela-induced hypocholesterolemic effect through its action on promotion of conversion of cholesterol to bile acids through activation of CYP7A1, PPAR up-regulation and down regulation of FAS, SREBP1c and HMG-CoAR in liver and adipose tissue. Our findings recommends the usage of karela as functional foods that has beneficial effects on human health.

## Abbreviations

ACO: Acyl CoA oxidase
cDNA: complementary deoxyribonucleic acid
CPT-1: Carnitine palmitoyltransferase-1
CYP7A1: Cholesterol 7α-hydroxylase
DAB: Diaminobenzidine
DEPC: Diethylpyrocarbonate
DNA: Deoxyribonucleic acid
EDTA: Ethylenediamine tetra acetic acid
FAS: Fatty acid synthase
GAPDH: Glyceraldhyde-3-phosphate dehydrogenase
GOT: Glutamate oxalacetate transaminase
GPT: Glutamate pyruvate transaminase
GSH: Reduced glutathione
GST: Glutathione-S-transferase
H and E: Hematoxylin and eosin
HMG-CoAR: 3-hydroxy-3-methylglutaryl coenzyme A reductase
LRH-1: Liver receptor homologue-1
LXRα: Liver X receptor-α
M-Mul V: Moloney Murine Leukemia Virus
NBF: Neutral buffered formalin
PEPCK: Phosphoenolpyruvate carboxykinase
PK: Pyruvate kinase
PPAR-α: Peroxisome proliferator-activated receptor alpha
PPAR-γ: Peroxisome proliferator-activated receptor gamma
RNA: Ribonucleic acid
RT-PCR: Reverse transcription polymerase chain reaction
SE: Standard error
SHP: Small heterodimer partner
SOD: Superoxide dismutase
SREBP-1c: Sterol responsible element binding protein-1c
TBE: Tris-borate-EDTA
TC: Total cholesterol
